# Interleukin-7 Plasma Levels in Human Differentiate Anorexia Nervosa, Constitutional Thinness and Healthy Obesity

**DOI:** 10.1371/journal.pone.0161890

**Published:** 2016-09-09

**Authors:** Natacha Germain, Odile Viltart, Anne Loyens, Céline Bruchet, Katia Nadin, Isabelle Wolowczuk, Bruno Estour, Bogdan Galusca

**Affiliations:** 1 Division of Endocrinology, Diabetes, Metabolism and Eating Disorders, CHU Saint-Etienne, Saint-Etienne, France; 2 TAPE (Eating Disorders, Addictions & Extreme Bodyweight) Research Group EA 7423, Saint-Etienne, France; 3 Univ. Lille, INSERM, CHU Lille, UMR-S 1172 - JPArc - Jean-Pierre AUBERT Research Center, Neurosciences and Cancer, F-59000, Lille, France; 4 Univ. Lille, CNRS, Inserm, CHU Lille, Institut Pasteur de Lille, U1019 - UMR8204 - CIIL - Center for Infection and Immunity of Lille, F-59000, Lille, France; CNRS, University of Strasbourg, FRANCE

## Abstract

**Introduction:**

Interleukin-7 (IL-7) is a cytokine involved in energy homeostasis as demonstrated in rodents. Anorexia nervosa is characterized by restrained eating behavior despite adaptive orexigenic regulation profile including high ghrelin plasma levels. Constitutional thinness is a physiological condition of resistance to weight gain with physiological anorexigenic profile including high Peptide YY plasma level. Healthy obesity can be considered as a physiological state of resistance to weight loss with opposite appetite regulating profile to constitutional thinness including low Peptide YY plasma level. No studies in IL-7 are yet available in those populations. Therefore we evaluated circadian plasma levels of IL-7 in anorexia nervosa compared to constitutional thinness, healthy obese and control females.

**Materials and Methods:**

10 restrictive-type anorexia nervosa women, 5 bingeing/purging anorexia nervosa woman, 5 recovered restrictive anorexia nervosa women, 4 bulimic females, 10 constitutional thinness women, 7 healthy obese females, and 10 normal weight women controls were enrolled in this cross-sectional study, performed in endocrinology unit and academic laboratory. Twelve-point circadian profiles of plasma IL-7 levels were measured in each subject.

**Results:**

24h mean IL-7 plasma levels (pg/ml, mean±SEM) were decreased in restrictive-type anorexia nervosa (123.4±14.4, p<0.0037), bingeing/purging anorexia nervosa (24.2±5.6, p<0.001), recovered restrictive anorexia nervosa (64.2±16.1, p = 0.01) and healthy obese patients (51±3.2, p<0.001) compared to controls (187.7±28.6). Bulimic patients (197.4±42.7) and constitutional thinness patients (264.3±35.8) were similar to controls.

**Conclusions:**

Low IL-7 is part of the adaptive profile in restrictive-type anorexia nervosa, confirming its difference with constitutional thinness. Healthy obesity, with low IL-7, is once again in mirror image of constitutional thinness with normal high IL-7.

## Introduction

Relation between body mass index, body composition and appetite regulation is complex. Anorexia nervosa (AN) and constitutional thinness (CT) are two conditions associated with low body weight. If AN is an eating disorder with undernutrition, CT is a physiological state of resistance to body weight gain exempt of undernutrition [1] [2]. Healthy obesity is characterized by a high body weight without any metabolic syndrome [3].

In AN, noticeable adaptive changes to food restriction in gut hormones in charge of appetite regulation and energy metabolism are now widely described. An overall orexigenic tone is indeed observed including high plasma level of ghrelin and 26RFa, normal plasma levels of neuropeptide Y (NPY), and low plasma level of leptin and alpha-melanocyte-stimulating hormone [4] [5] [6] [7] [8]. On the opposite, CT displays anorexigenic profile [2], with low-to-normal plasma levels of ghrelin and high plasma level of PYY associated with high postprandial surge of PYY and GLP-1 [2] [5]. Interestingly normal-low leptin in CT is associated with preserved menses compared to AN patients [5]. In obese patients, plasma levels of ghrelin are low and plasma levels of leptin are high, [9]. PYY and GLP-1 increment after meal are blunted in a mirror image of CT [10].

Besides gut hormones, immune system is also known to modulate appetite regulation through direct action on hypothalamus, especially by pro-inflammatory cytokines [11] [12] [13]. However, literature is often controversial about a potential dysregulation in cytokines network in AN [14]. Indeed TNFα was found normal or increased and not modified by body weight recovery [15] [16] [17]. IL-2, TGFβ2 and soluble IL-6 receptor were found decreased in AN [17]. IL-6 level was either normal or increased in AN and normalized after weight recovery [16] [17], while IL-1, IL-4, IL-10 and interferon gamma (INFγ) levels were comparable to controls [16] [17]. In obesity, high levels of pro-inflammatory cytokines are described in non-metabolically healthy subjects in particular in those with insulin resistance [18] [19].

IL-7 is a recently studied cytokine, first identified in the bone marrow and peripheral lymphoid organs [20], but finally mainly produced by non-hematopoietic cells including skin, liver, brain and white adipose tissue [21] [22]. IL-7 exerts pleiotropic effects, with both anti-inflammatory and pro-inflammatory role [23] [24]. Recent studies in rodents showed that the role of IL-7 might be extended to metabolism. Indeed, in mice, administration of exogenous IL-7 decreased food intake in situation of refeeding after fasting, by modulating pro-opiomelanocortin neurons’ activity [25]. Moreover fasting plasma levels of IL-7 was lately associated with insulin resistance in obese women with prediabetes [18] [19]. Oppositely, a recent study showed that IL-7 might protect rodents from obesity through regulation of adipose tissue via a lymphocyte-independent mechanism [26]. Those data are controversial partly due to heterogeneity of inflammatory state in obesity. Finally, plasma level of IL-7 has not been specifically yet evaluated in AN patients and CT, two populations with low BMI.

It is well known that appetite regulation and subsequent food intake present with a circadian profile. Thus, one-time point assessment approach of appetite regulation or cytokines profiles in eating disorders or extreme body weight could explain most of mentioned above controversial results. Circadian evaluation of appetite regulation in eating disorders strengths discrepancies between studied groups and reveal blunted rhythm [4] [6]. Along with that, immune systems activity and cytokines levels in particular were shown to display daily rhythms in parallel with hypothalamic pituitary axis hormonal pattern [27] [28].

According to all those elements, this study was thus designed to explore IL-7 circadian plasma levels over 24 hours in eating disorder’s patients, CT, and healthy obese patients compared to a control group.

## Materials and Methods

### Ethics

Study protocol was conducted according to the principles of the declaration of Helsinki and approved by the local Human Research Ethic Committee of the Saint- Etienne University Hospital Center (France). All subjects gave a written informed consent for biological assessment.

### Subjects

The study included fifty one women in 7 groups of Caucasian women: anorexia nervosa restrictive type (AN-R), anorexia nervosa restrictive type after recovery (AN-R rec), anorexia nervosa bulimic type (AN-BP), constitutional thinness (CT), bulimia nervosa (BN), healthy obese (OB), and control subjects (C).

Fifteen AN women were recruited during their first hospitalization, before any treatment or intervention. They met DSM-IV criteria [1]. They displayed Body Mass Index (BMI) < 17 kg/m^2^. None of them were under oral contraception and they all presented with amenorrhea for at least 6 months. 10 were restrictive type (AN-R) and 5 were bulimic-type (AN-BP). Five AN-R were evaluated a second time after weight and menses recovery (AN-R rec).

Four bulimia nervosa patients (BN), with normal body weight (BMI between 19 and 25 kg/m^2^) and no amenorrhea were selected according to DSM-IV criteria. They used no oral contraception and presented one episode of bingeing/purging per day.

Ten CT subjects were recruited based on the following criteria: BMI < 17 kg/m^2^, stable weight throughout the growth period, physiological menstruations without oral contraception and a stated desire for weight gain.

Seven metabolically healthy obese (OB) women were enrolled using the following criteria: BMI > 30 kg/m^2^, no metabolic syndrome and physiological menstruations without oral contraception.

Ten normal body weight women were selected as controls (mean BMI between 19 and 25 kg/m^2^) with similar age range (18–38 years) and without any eating disorders.

For CT, BN, OB and controls, all samples were collected during the follicular phase of cycle. None of the subjects had documented chronic or congenital disease, smoking, drugs intake and none of them were taking any medication.

### Sampling

Blood sampling was carried out during an inpatient period of 24-hour. After collection, venous samples were immediately centrifuged and plasmas were aliquoted and kept frozen at -80°C until further analysis. In order to phenotype specifically each group of patients, IGF-1, 17β-estradiol, FSH, LH, testosterone, free T3 were measured at 0800h after an overnight fast. Aspartate Transaminase (AST) and Alanine Transaminase (ALT) Gamma Glutamic Transferase (GGT), Alkaline Phosphatase (ALP), insulin, blood glucose and HbA1c were also measured in obese patients only. HOMA and QUICKI index were calculated in OB population [29]. Leptin and cortisol were sampled in 6 time-points over 24-hour (0800h, 1200h, 1600h, 2000h, 2400h and 0400h). IL-7 was assessed over 12 time-points (0700h, 0800h, 0900h, 1000h, 1200h, 1300h, 1400h, 1600h, 2000h, 2400h and 0400h). Standardized meal were proposed at 0815h (400 kcal), 1215h (800 kcal) and 1915h (800 kcal) and certainly eaten in CT, BN, OB and controls group. Snacks were not allowed in addition to the standardized meals.

### Body Composition and Energy Metabolism Measurements

Total body composition was assessed by Dual Energy X-ray Absorptiometry (DXA) to quantify fat mass and lean mass (LUNAR DPX-L, intra assay coefficient of variation <1%) [30].

### Plasma Assays

Plasma levels of IL-7 were determined by a two-step enzyme-linked immunosorbent assay (ELISA) using recombinant human IL-7 to establish the standard curve (from 6.25 to 800 pg/mL) (reference: 207-IL, R&D Systems, Minneapolis, US), anti-human IL-7 antibody (Ab) as capture Ab (reference: 554493, Pharmingen, San Diego, USA) and biotinylated anti-human IL-7 Ab as detection Ab (reference: 554494, Pharmingen, San Diego, USA). Chromogenic revelation was done using streptavidin conjugated to horseradish peroxidase (HRP) (reference: 016-030-084 Jackson ImmunoResearch, Montluçon, France) and tetramethylbenzidine (TMB) substrate (Thermofisher, IllKirch, France). Plates were read at 450nm and 595nm (Multiskan Ascent, Thermo Labsystems, Cambridge, UK). Samples were diluted 1:3. Assessment of other hormonal parameters was previously described [31]. Leptin was measured by RIA (Nichols Institute Diagnostics, San Juan Capistrano, CA, USA), normal values for a normal BMI (18–25) 3.7–11.1 mg / L); Cortisol by RIA (cortisol, Immunotech), coefficients of variation within and between test 7% and 8% respectively, detection limit 10 nmol / L, normal values (107 to 310 nmol / L); IGF1 by electrochemiluminescence on COBAS PLC; 17β estradiol by RIA (Dia Sorin, France), normal values during the follicular phase 30–50 ng / L; FSH and LH by IRMA (Beckman Coulter), normal values during the follicular phase 1.8–10.5 IU / L and 0.5–5 IU / L respectively; Testosterone by RIA (Beckman), extraction and chromatography, normal range 7–65 ng / dL; Free T3 by RIA (Beckman), normal values 2.5–5.8 pmol / L.

### Statistical Analysis

All values are expressed as mean ± SEM (standard error of the mean). Intergroup comparison of one-time measured parameters was performed by ANOVA followed by a post-hoc test (Fisher’s Protected Least Significant Difference) when significant. Two-factor repeated measures ANOVA (group x time) test followed by an adapted post-hoc analysis was used to evaluate inter- and intra-group differences of IL-7, cortisol and leptin plasma levels. Correlations between 24-hour mean IL-7 plasma levels and 24-hour mean leptin, and cortisol plasma levels, as well as BMI, fat mass percentage and age were also evaluated. Statistical significance was set at p< 0.05. All statistical analyses were performed with the StatView 4.5 software (Abacus Concepts, Inc., Palo Alto, CA) and GraphPad Prism 5.0 software (GraphPad Software, San Diego, CA).

## Results

### Population’s Characteristics (Table 1)

**Table 1:** Population characteristics.

Parameters	Controls (n = 10)	AN-R (n = 10)	AN-R rec (n = 5)	AN-BP (n = 5)	BN (n = 4)	CT (n = 10)	OB (n = 7)
**Age (years)**	22.7 ± 0.5	21.6 ± 1.5	21.8 ± 2	21.8 ± 2.4	24.2 ± 4.0	20.6 ± 2.1	27 ± 2.1*
**Height (m)**	1.64 ± 0.02	1.62 ± 0.03	1.61 ± 0.04	1.65 ± 0.03	1.64 ± 0.03	1.61 ± 0.02	1.65 ± 0.04
**Weight (kg)**	57.5 ± 1.2	39.2 ± 1.3 *^£^^¢^^#^	54.7 ± 6.1°^#^	41.9 ± 3.5 *	53.6 ± 2.6°^#^	41.1 ± 1.2 *^#^	127.8 ± 13*
**BMI (kg/m**^**2**^**)**	21.4 ± 0.5	15.1 ± 0.8 *^$^^¢^^#^	20.9 ± 1.7°^#^	15.4 ± 1.2 *^£^^¢^^#^	19.8 ± 0.6°^#^	15.9 ± 0.3 *^#^	46.2 ± 3.4*
**Fat mass (%)**	30.0 ± 2.4	15.8 ± 4.1 *^£^	31.2 ± 3.3	15.6 ± 4.7 *^£^	26.3 ± 3.5	23.9 ± 2.1	**―**
**Mean leptin (μg/L)**	15.8 ± 2.3	1.7 ± 0.4 * ^£^^¢^°^#^	14.5 ± 5°^#^	2.7 ± 0.9 *^£^^#^	14.2 ± 2.4 ^#^	5.5 ± 0.9 ^#^	64.5 ± 16.4 *
**Mean cortisol (nmol/L)**	285.6 ± 26.5	461.5 ± 33.1 *^£^°^¢^	296.9 ± 45.1	371.2 ± 27.6	287.0 ± 13.0	246.5 ± 15.2	**―**
**IGF1 (ng/mL)**	245.3 ± 16.7	123.9 ± 20.7 *°^¢^^£^	243.0 ± 30.8	162.6 ± 41.7 *	232.5±17.6	290.3 ± 42.1	171 ± 22 *
**17βEstradiol (pg/mL)**	41.9 ± 18.3	7.8 ± 1.8 *°^£^	32.4 ± 12.8	13.8 ± 4.6 *°	31.0 ± 20.2	40.1 ± 5.5	**―**
**FSH (mUI/mL)**	6.1 ± 1.2	2.1 ± 0.6 *°^£^	4.8 ± 1.2	4.5 ± 0.6 ^$^	4.0 ± 1.2	6.3 ± 0.6	**―**
**LH (mUI/mL)**	10.10 ± 4.10	0.67 ± 0.35 *^¢^°^£^	17.56 ± 5.94°	2.12 ± 1.00 *°	4.50± 2.43 *	9.70 ± 0.96	**―**
**Testosterone (ng/dL)**	75.3 ± 13.2	63.4 ± 12.1	49.4 ± 4	44.4 ± 10.5	42.3 ± 4.3	50.8 ± 8.5	**―**
**Free T3 (ng/L)**	4.3 ± 0.2	3.0 ± 0.3 *^#^°^¢^^£^	4.1 ± 0.4 ^#^	2.74 ± 0.3 *^#^°^¢^^£^	4.27 ± 0.2 ^#^	4.6 ± 0.19 ^#^	5.4 ± 0.3 *

Data are expressed as mean ± SEM. AN-R is for restrictive type anorexia nervosa patients, AN-R rec is for AN-R patients after weight recovery, AN-BP is for bulimic type AN patients, BN is for bulimia nervosa patients, CT is for constitutional thinness, OB is for healthy obese patients. Statistics:

*: p<0,05 vs. controls;

^$^: p<0,05 vs AN-R;

^£^: p<0,05 vs AN-R rec;

^¢^: p<0,05 vs AN-BP;

°: p<0,05 vs CT and

^#^: p<0,05 vs OB.

**Fig 1:**
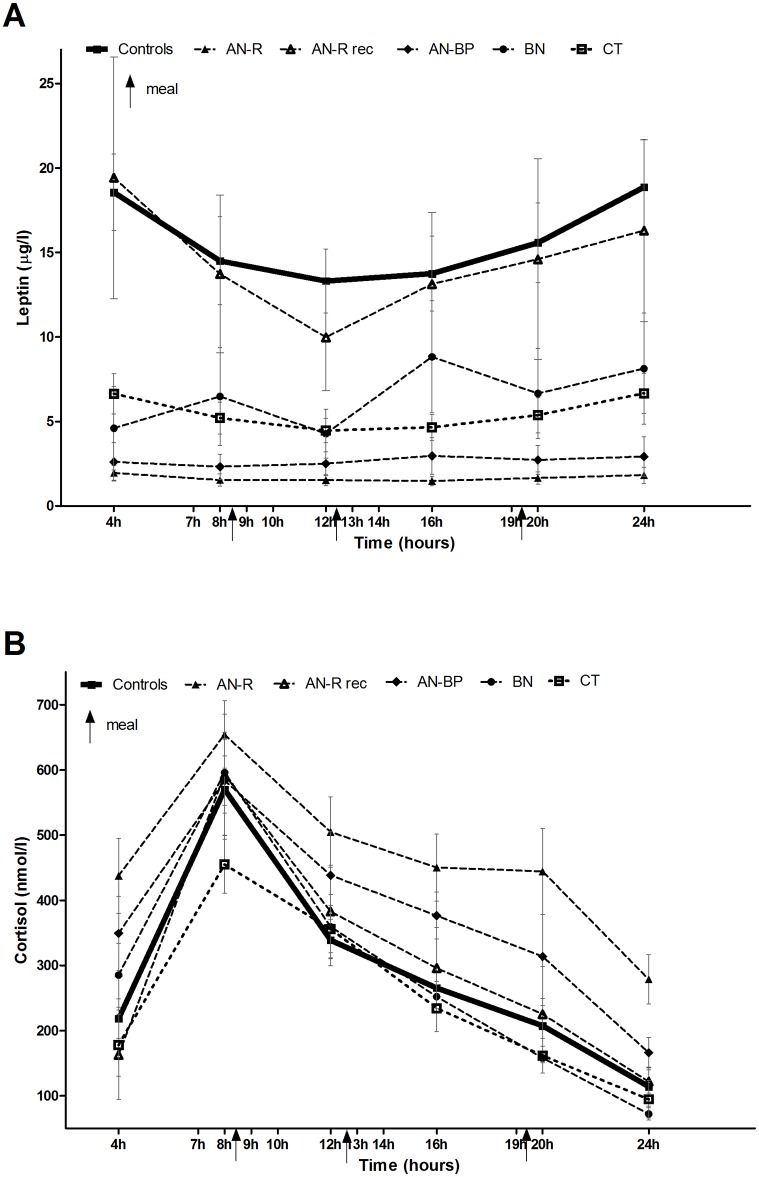
Six-point circadian profiles of Leptin (A) and Cortisol (B). AN-R is for restrictive type anorexia nervosa patients, AN-R rec is for AN-R patients after weight recovery, AN-BP is for bulimic type AN patients, BN is for bulimia nervosa patients, CT is for constitutional thinness. Arrows indicate meals schedule. Circadian profile of cortisol plasma level was not evaluated in OB.

The groups were comparable in age and height except for obese women who were significantly older than controls (27.0 ± 2.1 years vs. 22.7 ± 0.5 years, p = 0.03). BMI of AN-R, AN-BP and CT was significantly lower compared to controls (p<0.001) while BMI of OB was significantly higher compared to controls (p<0.001). Fat mass percentage and leptin plasma levels were significantly decreased in AN-R compared to controls (p = 0.047 and p<0.001 respectively), but also compared to CT (p = 0.05 and p = 0.002 respectively) and to AN-R rec patients (p = 0.002 and p = 0.0001 respectively). Leptin was significantly increased in OB patients compared to controls (p = 0.001). In OB patients, mean AST was at 22±2.1 mU/l, ALT at 19±6.4 mU/l, GGT at 36±8 U/L, ALP at 58±10 U/L, HbA1c was at 5.5±0.4%, HOMA index was at 1.7±0.5 and QUICKI index was at 0.6±0.05 all values in the normal range. AN patients presented with impaired hypothalamic pituitary axis regulation: 24-hour mean cortisol was significantly higher compared to controls (p<0.001) and CT (p<0.001). IGF-1 was significantly lower compared to controls (p<0.001 for AN-R and p = 0.04 for AN-BP). Free T3 was reduced in both types of AN patients when compared to controls (p = 0.001 and p<0.001) and normalized after weight recovery. AN patients presented with hypogonadotropic hypogonadism with significantly decreased LH, FSH and 17βEstradiol plasma levels compared to controls (p = 0.03, p = 0.009 and p = 0.01 respectively) and also normalized after body weight recovery. On the opposite, CT displayed no hormonal abnormalities when compared to controls including 24-hour mean cortisol, IGF-1 and Free T3. OB patients displayed no metabolic syndrome, but their IGF-1 was significantly decreased compared to controls (p = 0.02). Daily profiles of plasma cortisol and leptin are presented in fig 1, and showed conserved circadian rhythm of cortisol in all groups and blunted circadian leptin pattern in AN-R patients.

### 24-Hour Mean Plasma Levels of IL-7 (Fig 2)

**Fig 2:**
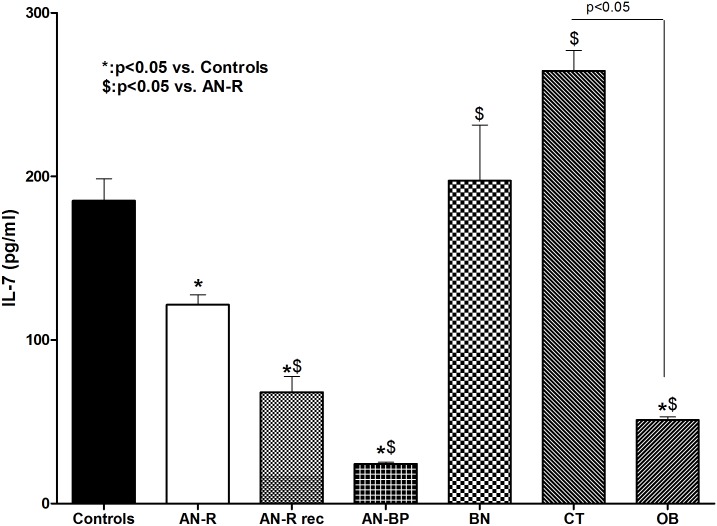
24-hour mean level of IL-7. Data are expressed as mean ± SEM. AN-R is for restrictive type anorexia nervosa patients, AN-R rec is for AN-R patients after weight recovery, AN-BP is for bulimic type AN patients, BN is for bulimia nervosa patients, CT is for constitutional thinness, OB is for healthy obese patients. Statistics: *: p<0.05 vs. controls.

24-hour mean levels of IL-7 were significantly lower in each AN patients subtypes when compared to controls (123.4±14.4 pg/ml in AN-R *vs*. 187.7±28.6 pg/ml in controls, p = 0.037; 64.2±16.1 pg/ml in AN-R rec, p = 0.01 *vs*. controls; 24.2±5.6 pg/ml in AN-BP, p<0.001 *vs*. controls). IL-7 was also significantly lower in AN-BP than in AN-R (p<0.01) and significantly decreased after recovery in AN-R (p = 0.03). IL-7 in BN (197.4±42.7 pg/ml) did not differ from that of controls but was significantly increased compared to AN-R (p = 0.04). IL-7 in CT was also similar to controls (264.3±35.8 pg/ml, p = 0.1) and significantly increased when compared to AN-R (p<0.01). Oppositely IL-7 was significantly decreased in OB compared to controls (51±3.2 pg/ml, p<0.001), AN-R (p<0.01) and CT (p = 0.01).

### Twelve-Point Circadian Profile of Plasma Levels of IL-7 (Fig 3)

**Fig 3:**
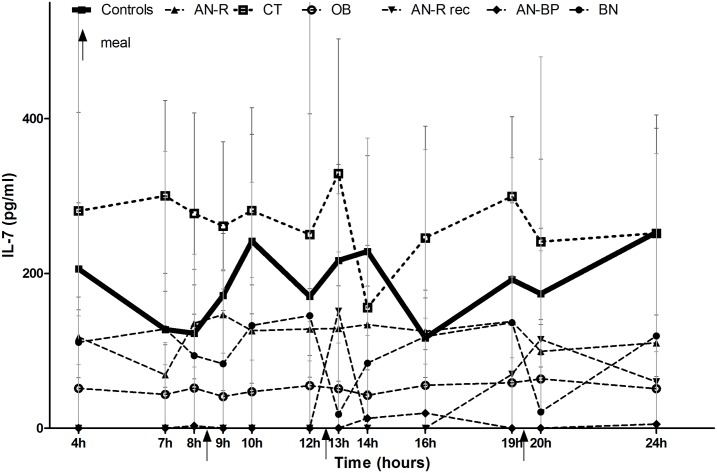
Twelve-point circadian profiles of IL-7 in all groups of the study. AN-R is for restrictive type anorexia nervosa patients, AN-R rec is for AN-R patients after weight recovery, AN-BP is for bulimic type AN patients, BN is for bulimia nervosa patients, CT is for constitutional thinness, OB is for healthy obese patients. Arrows indicate meal schedule.

Statistical analysis of circadian values showed no variation during meal schedules in all groups. Lower values were found for day time-points, when compared to evening ones, in AN-R (p = 0.04 between 9000h and 2000h), in CT (p = 0.05 between 0700h and 2000h and between 1400h and 2000h; p = 0.01 between 1400h and 2000h) and in OB (p = 0.02 between 0900h and 1600h).

Point by point comparisons between groups showed a trend in lower values for OB compared to CT at 0700h, 0900h and 1900h (p = 0.08). BN showed significantly higher values of IL-7 than OB during the 24-hour at 0700h (p<0.001), 0900h (p<0.001), 1000h (p = 0.035), 1200h (p = 0.002), 1400h (p = 0.002), 1600h (p<0.001) and 2400h (p<0.001). At 1200h and 2000h, AN-R exhibited significantly lower IL-7 values compared to normal weight BN patients (p = 0.034 and p = 0.03).

### Correlations Analysis

No correlation between mean plasma IL-7 and fat mass, mean leptin levels, BMI, mean cortisol or age of the participants were found in any of the groups except for mean leptin which positively correlated with IL-7 (r = 0.9, p = 0.008) in control group (S1 Table).

## Discussion

The present study is the first published report evaluating IL-7 circadian profile in humans in several categories of body weight and eating disorders. It shows two main findings: a possible biphasic circadian pattern of IL-7 and significant differences of IL-7 mean values between the studied groups.

This study reveals first a circadian profile of IL-7 in humans. Although IL-7 has been suggested to be anorectic in fasted rodents, no post prandial surge was individualized in our study, as it has been seen in short-term anorexigenic hormones such as GLP-1 and PYY [2].

However, we found a possible biphasic circadian profile for IL-7 in AN-R, CT and OB, with an increase during the second part of the nycthemeron compared to the morning. This is in line with a study that reported higher IL-7 levels during sleep conditions compared to wakefulness period [32]. Interestingly, we previously showed that orexigenic 26 RFa displayed an opposite circadian profile in AN-R, with higher levels in the first part of the day than in the evening in controls [6]. Il-7 pattern should be interpreted with regards to hypothalamic pituitary axis circadian hormonal pattern linked to clock genes [33]. Indeed circadian rhythm of plasma cortisol, involved in cytokines rhythm regulation [28], is preserved in all kind of studied patients, even in AN-R patients [34], whereas the possible biphasic pattern was only found in AN-R, CT and OB. Leptin, a biphasic and anorexigenic peptide, was found blunted in AN-R as previously reported [35], and preserved in all the other groups. This possible biphasic pattern, found in some studied groups, needs to be confirmed before assuming any physiological conclusions.

The main finding of this study is the significant differences of IL-7 mean values between the studied groups.

IL-7 was low in all underweight subtypes of AN patients when compared to controls levels. However, high level of IL-7 has been found in undernourished HIV patients with major immune system alterations and decreased appetite [36]. Studies on immune system in AN are controversial while AN is not associated with major immune system impairment in literature [14]. Finally, those studies have been done on one time-point sampling contrary to our present circadian analysis, which could partly explain the discrepancy. Moreover, these data also strengthen previous results showing an orexigenic profile in AN, that might counteract the current restrictive eating behavior, in addition to already reported high ghrelin [31], low PYY [5], low leptin, and high 26Rfa [6]. This low level of IL-7 could be interpreted as a consequence of an adaptation mechanism in order to face the absence of food intake. Decreased IL-7 in AN patients after weight recovery, regardless other hormones such as ghrelin, leptin, free T3 or IGF1, was surprising [5]. However this is in line with previous studies on TNFα showing no return to normal level after weight recovery in AN patients [15] [16]. Taken together, this suggests other factors than weight and/or eating behavior to regulate cytokines in AN. Interestingly, IL-7 was significantly different between the subtypes of AN patients. Bingeing/purging was found associated with decreased IL-7 plasma levels in AN-BP but not in normal weight BN. In our previous study, ghrelin was found decreased in both AN-BP and normal weight BN [37]. We could therefore assume that eating behavior is not the only regulating factor of IL-7.

CT subjects displayed similar levels of IL-7 compared to controls, but significantly increased compared to each type of AN patients. This is another biomarker that differentiates CT from AN, and could be helpful for differential diagnosis. These data are also in line with our previous results in CT showing a constitutive/physiological sub-anorexigenic profile, along with low-to-normal ghrelin, high PYY [5] and increased post prandial GLP1 and PYY surges [2].

Interestingly metabolically healthy obese patients presented with low IL-7 plasma levels when compared to controls. This result can be added within the complex immuno-metabolic profile characterizing obesity [38]. MRNA of IL-7 in omental fat tissue and IL-7 secretion in those adipocytes were previously studied along with other cytokines in humans. IL-7 was found overexpressed in adipocytes of obese insulin resistant patients compared to normal weight ones [19]. IL-7 was also shown increased in obese diabetic patients compared to obese non-diabetic ones [18] and related to insulin resistance [39]. However, a pilot study evaluated IL-7 expression in blood cells of obese women with metabolic syndrome and found no difference with controls [40]. All these data suggest that IL-7 could possibly be over secreted under inflammation and insulin resistance conditions. In our study, obese subjects were selected with metabolic healthy phenotype, including no insulin resistance and no diabetes, which could explain why IL-7 was low and not increased by inflammation. On the other hand, Lucas et al. previously reported a protective role of IL-7 on weight gain [26]. Moreover, transgenic mice overexpressing IL-7 showed a decreased fat mass. Indeed, in induced-obesity animal models, IL-7 administration prevented from weight gain, by a mechanism independent of lymphocyte cells in which IL-7 could act as a negative regulatory factor of adipocytes differentiation and a positive regulator of lipolysis [41]. Here again, literature is highly controversial and confusing, mainly due to heterogeneity of inflammation in obesity, one-time sample approach and mixed human and rodents studies. It is therefore too early to speculate on a possible role of this low IL-7 value in healthy obesity. All these together, we describe here another biological markers showing opposition between CT and healthy obesity.

This study raises some limitations. The small number of patients included in each group is counteracted by the very tight phenotyping of each one. IL-7 values in our study are different from what was already published in literature in humans [42]. But our study is the first to present a circadian profile of IL-7 in a control population, which may explain this difference. The assay was done once for all groups in order to limit variations and allow comparisons between groups. We did not assess pro or anti-inflammatory markers. Thus, it would be of interest to compare IL-7 along with other cytokines in these same populations in order to discriminate pro or anti-inflammatory effect of IL-7. Finally, this work focused only on women and it would be also relevant to investigate similar groups of men to improve our knowledge on the role of estrogens/testosterone in IL-7 regulation. Indeed some authors described specific characteristics of leptin circadian profile with regards to gender [43].

## Conclusion

The present study is the first published report evaluating IL-7 circadian profile in human in several categories of body weight and eating disorders. IL-7 showed no prandial rhythm but might have a biphasic circadian pattern in some groups that needs to be confirmed. Plasma level of IL-7 is low in anorexia nervosa suggesting that IL-7 has no role in resistance to orexigenic signal but is another peptide taking part of the adaptive orexigenic profile in order to counteract the food restriction. Plasma level of IL-7 is normal-high in CT, confirming once again differential diagnosis between AN and CT. Finally opposite levels of IL-7 between CT and OB is, along with other peptides, another data suggesting that constitutional thinness is the mirror image of healthy obesity.

## Supporting Information

S1 Table Correlation between 24h mean IL-7 plasma level and fat mass percentage, 24h mean leptin plasma level, body mass index, 24h mean cortisol and age in all groups of patients. Data are expressed as p-value. AN-R is for restrictive type anorexia nervosa patients, AN-R rec is for AN-R patients after weight recovery, AN-BP is for bulimic type AN patients, BN is for bulimia nervosa patients, CT is for constitutional thinness, OB is for healthy obese patients.
(DOCX)
