# Supplementary material for: Interleukin-7 Plasma Levels in Human Differentiate Anorexia Nervosa, Constitutional Thinness and Healthy Obesity
Source: PLoS One. 2016 Sep 9;11(9):e0161890. doi: 10.1371/journal.pone.0161890 (PMC5017702; doi:10.1371/journal.pone.0161890)
Supplement: S1 Table — Data are expressed as p-value. AN-R is for restrictive type anorexia nervosa patients, AN-R rec is for AN-R patients after weight recovery, AN-BP is for bulimic type AN patients, BN is for bulimia nervosa patients, CT is for constitutional thinness, OB is for healthy obese patients. (DOCX) [file pone.0161890.s001.docx]

|  | **Controls (n=10)** | **AN-R (n=10)** | **AN-R rec (n=5)** | **AN-BP (n=5)** | **BN (n=4)** | **CT (n=10)** | **OB (n=7)** |
| --- | --- | --- | --- | --- | --- | --- | --- |
| **Fat mass percentage** | 0.99 | 0.55 | 0.99 | 0.45 | 0.75 | 0.80 | 0.99 |
| **24h mean Leptin** | **0.0078** | 0.36 | 0.99 | 0.73 | 0.77 | 0.84 | 0.10 |
| **Body mass index** | 0.12 | 0.44 | 0.99 | 0.86 | 0.78 | 0.84 | 0.13 |
| **24h mean Cortisol** | 0.57 | 0.29 | 0.99 | 0.37 | 0.61 | 0.36 | 0.99 |
| **Age** | 0.42 | 0.61 | 0.99 | 0.81 | 0.28 | 0.48 | 0.84 |

**S1 Table:** **Correlation between 24h mean IL-7 plasma level and fat mass percentage, 24h mean leptin plasma level, body mass index, 24h mean cortisol and age in all groups of patients.** Data are expressed as p-value. AN-R is for restrictive type anorexia nervosa patients, AN-R rec is for AN-R patients after weight recovery, AN-BP is for bulimic type AN patients, BN is for bulimia nervosa patients, CT is for constitutional thinness, OB is for healthy obese patients.
